# Evaluation of in-vivo and in-vitro binding property of a novel PET tracer for CSF1R imaging and comparison with two currently-used CSF1R-PET tracers

**DOI:** 10.21203/rs.3.rs-6194254/v1

**Published:** 2025-03-20

**Authors:** Xiyan Rui, Yuzhou Ding, Nailian Zhang, Xinran Zhao, Chie Seki, Tomoteru Yamasaki, Masayuki Fujinaga, Ming-Rong Zhang, Jun Qian, Bin Ji, Rong Zhou

**Affiliations:** Tongji University Affilliated Yangpu Hospital: Shanghai Yangpu District Central Hospital; Fudan university; Tongji University Affilliated Yangpu Hospital: Shanghai Yangpu District Central Hospital; Fudan university; Tongji University Affilliated Yangpu Hospital: Shanghai Yangpu District Central Hospital; Fudan university; Tongji University Affilliated Yangpu Hospital: Shanghai Yangpu District Central Hospital; Fudan university; Advanced Advanced Neuroimaging Center, National Institutes for Quantum Science and Technology; Department of Advanced Nuclear Medicine Sciences,Institute for Quantum Medical Science, National Institutes for Quantum Science and Technology; Department of Advanced Nuclear Medicine Sciences,Institute for Quantum Medical Science, National Institutes for Quantum Science and Technology; Department of Advanced Nuclear Medicine Sciences,Institute for Quantum Medical Science, National Institutes for Quantum Science and Technology; Fudan University School of Pharmacy; Fudan University School of Pharmacy; Tongji University Affilliated Yangpu Hospital: Shanghai Yangpu District Central Hospital

**Keywords:** Positron emission computed tomography (PET), Autoradiography, Colony-stimulating factor 1 receptor (CSF1R), [11C]CPPC, [11C]o-aminopyridyl alkynyl derivative

## Abstract

**Background:**

Colony-stimulating factor 1 receptor (CSF1R) is a promising imaging biomarker for neuroinflammation or tumor-associated macrophages. However, existing positron emission tomography (PET) tracers for CSF1R imaging commonly are suffering from limited specificity or sensitivity.

**Results:**

We have performed ^11^C-labeled radiosynthesis of compound FJRD (3-((2-amino-5-(1-methyl-1*H*-pyrazol-4-yl)pyridin-3-yl)ethynyl)-*N*-(4-methoxyphenyl)-4-methylbenzamide) with excellent affinity for CSF1R and evaluated its *in*-*vivo* and *in*-*vitro* binding properties. PET images of [^11^C]FJRD show low brain uptake and specific binding in the living organs except kidneys in the normal mice and rats. *In*-*vitro* autoradiographs show high-level specific binding in all investigated organs including brain, spleen, liver, kidneys and lungs when used self-blocking. Addition of cold CPPC partially blocked *in*-*vitro* [^11^C]FJRD binding in the various organs with blocking effects from 9 to 67%, and other two CSF1R inhibitors, GW2580 and BLZ945, showed minimal blocking effect, suggesting unignorable off-target binding in these organs. Meanwhile specific bindings of [^11^C]CPPC and [^11^C]GW2580 were faint in the mouse organs except [^11^C]CPPC specific binding detectable in the spleen.

**Conclusions:**

These results suggest [^11^C]FJRD as a potential CSF1R-PET tracer for more sensitively detecting CSF1R compared to [^11^C]CPPC and [^11^C]GW2580. However, high-level off-target binding requires further improvement in specificity for CSF1R imaging.

## Background

Colony-stimulating factor 1 receptor (CSF1R) is a tyrosine kinase expressed on macrophages in the peripheral tissues and microglia in the central nervous system (CNS) ^1–2^. Growing evidence highlights the pivotal roles of microglia and macrophages in driving neuroinflammation and establishing an immunosuppressive tumor microenvironment, both of which contribute to the progression of neurological disorders and tumor development^3–4^. Considering that the survival and proliferation of microglia and macrophages rely on the CSF1R signaling pathway, several CSF1R inhibitors have been developed as potential therapies for neurological disorders and cancer^5^. These treatments are either depending on depleting inflammatory microglia from the brain^2^, or reprogramming of microglia and macrophages from an immunosuppressive into an anti-tumor phenotype^6^. Therefore, non-invasive detection of microglia and macrophages through CSF1R imaging is crucial for monitoring spatiotemporal changes and enabling early medical intervention at the prodromal stages of these diseases. Positron emission tomography (PET), as a non-invasive imaging modality, is well-suited for serving as a valuable bridge between preclinical and clinical applications^7–8^. Several CSF1R-PET tracers, including [^11^C]CPPC and [^11^C]GW2580, have been developed to visualize activated microglia in the brains of rodents, non-human primates and human subjects with neuroinflammation^9^. *In-vitro* autoradiography and binding assays using [^3^H]CPPC revealed its limited ability to detect CSF1R associated with neurological disorders. Apart from the spleen, minimal specific binding is observed in normal organs such as the heart, liver, brain, kidneys, and lungs^10^. [^11^C]GW2580 exhibits a high level of non-specific binding, which further hinders its ability to achieve sensitive detection ^6^. Recently, researchers have reported a seria of *o*-aminopyridyl alkynyl derivatives with high affinity for CSF1R^11^. An *et al*. developed an ^18^F-labeled *o*-aminopyridyl alkynyl derivative, [^18^F]4 (3-((2-amino-5-(1-methyl-1*H*-pyrazol-4-yl)pyridin-3-yl)ethynyl)-*N*-(3-(2-fluoroethoxy)phenyl)-4-methylbenzamide), for CSR1R imaging and demonstrated a 25% increase in brain uptake in lipopolysaccharide-treated mice compared to normal controls^12^. This suggests that [^18^F]4 holds potential as an imaging agent for CSF1R. However, the specificity and superiority of *o*-aminopyridyl alkynyl derivatives as CSF1R-PET tracer remains unclear due to the lack of data on *in-vitro* binding properties and comparison with other CSF1R imaging tracers. To address such concerns, in this study, we conducted the ^11^C-labeled radiosynthesis of FJRD, a reported *o*-aminopyridyl alkynyl derivative with high affinity for CSF1R (IC_50_ = 1.4 nM) ^11^, and performed *in*-*vitro* and *in*-*vivo* evaluation in normal rodents. Additionally, we compared its *in-vitro* binding properties with two currently-used CSF1R-PET tracers, [^11^C]CPPC and [^11^C]GW2580.

## Results

### Chemical synthesis and radiolabeling

**Figure 1:**
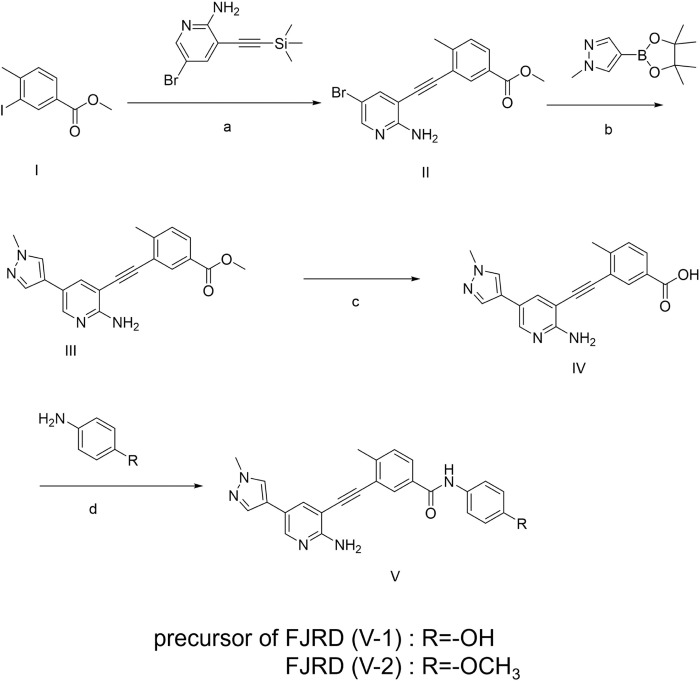
Chemical synthesis route of V-2 (FJRD) and V-1 (precursor for radiolabeling): a. Pd(PPh_3_)_2_Cl_2_, CuI, CsF, Et3N, react, 6 h, 88.2%; b. Pd(OAc)_2_, X-phos, K_2_CO_3_, THF, 6 h, 80%; c. LiOH·H_2_O, 12 h, 94%; d. HATU, DIPEA, 4h, 79.6%.

The synthetic pathways leading to compound **V-2** (FJRD) and **V-1** (precursor for radiolabelling) are depicted in Fig. 1. The synthetic sequence began with commercially available halogenated heteroaromatic compounds, which undergo a Sonogashira cross-coupling reaction with ethynyltrimethylsilane to yield intermediates **II** (methyl 3-((2-amino-5-bromopyridin-3-yl)ethynyl)-4-methylbenzoate). The intermediate **II** was then coupled with a boronic acid ester to produce the key intermediate **III** (Methyl 3-((2-amino-5-(1-methyl-1*H*-pyrazol-4-yl)pyridin-3-yl)ethynyl)-4-methylbenzoate). The hydrolysis of the methyl ester in intermediate **III** yields intermediate **IV** (3-((2-Amino-5-(1-methyl-1*H*-pyrazol-4-yl)pyridin-3-yl)ethynyl)-4-methylbenzoic acid), which was subsequently condensed with various substituted anilines to complete the synthesis.

**Figure 2:**
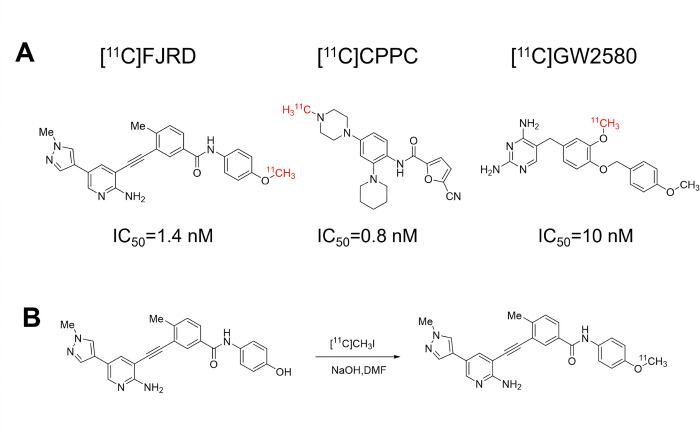
Chemical structures of CSF1R-PET tracers used in the present study and radiosynthesis of [^11^C]FJRD. (A) The affinities of [^11^C]FJRD^11^, [^11^C]CPPC^18^ and [^11^C]GW2580^9^ for CSF1R from the previous publications. (B) [^11^C]FJRD was radiosynthesized by one-step reaction of precursor and [^11^C]NaI.

The chemical structures of three PET tracers used in the present study and radiolabeling reaction of [^11^C]FJRD were shown in Fig. 2. [^11^C]FJRD was radiosynthesized with a 36% radiochemical yield based on [^11^C]CO_2_ (decay-corrected to the end of irradiation), with enough radioactivity and dependable quality for evaluation experiment. The total synthesis time was averaged to be 45 min from the end of the irradiation. The radiochemical purity of [^11^C]FJRD was greater than 90%, and the molar activity was approximately 120 GBq/μmol. The radiochemical purity of the final product solution was higher than 99% at the end of synthesis, with no significant peaks from impurity observed on the HPLC chromatogram. The radiochemical stability of [^11^C]FJRD was stable at least at 90 minutes after the formulated product solution was stored at 25°C, based on the evaluation of the radiochemical purity using HPLC.

### *In*-*vivo* whole body and brain imaging of [^11^C]FJRD in rodents

**Figure 3:**
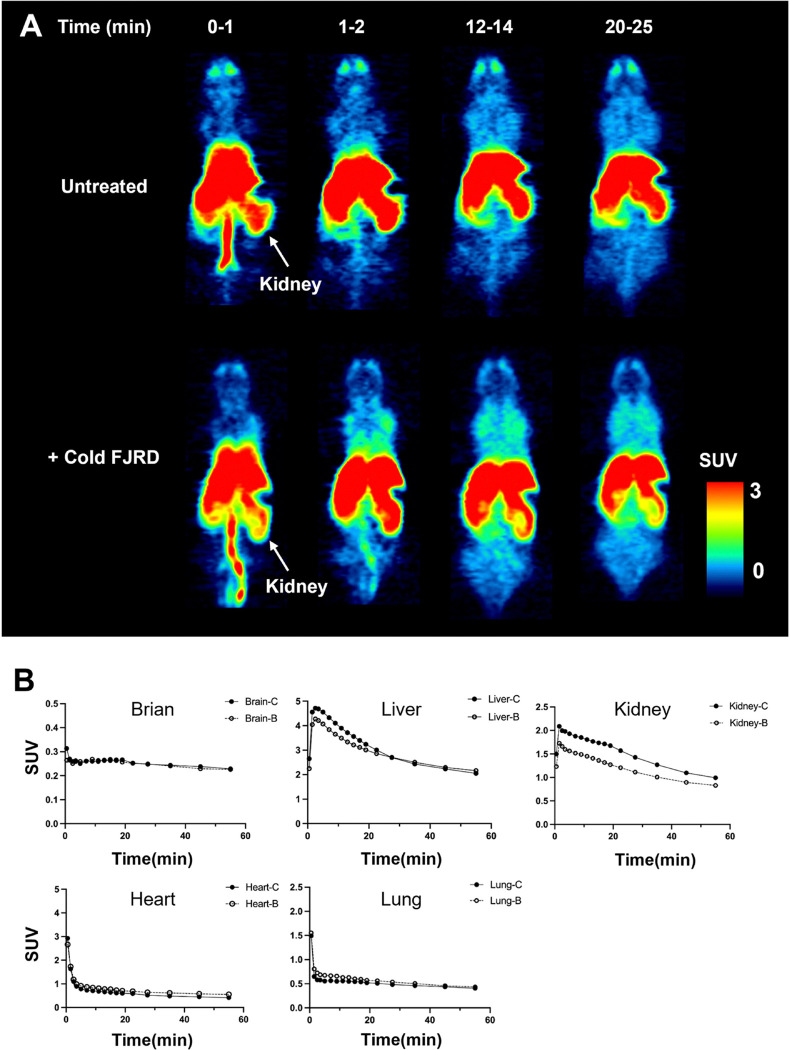
Whole body PET imaging with [^11^C]FJRD in mice. (A) Summation PET images of [^11^C]FJRD (0–1, 1–2, 12–14 and 20–25 min from left to right) with or without pretreatment of cold FJRD (1 mg/kg) as indicated. (B) Time-activity curves in the brain, heart, lung, liver, and kidneys. Data are expressed as SUV. -C: contro (no cold FJRD pretreatment); -B: block (cold FJRD pretreatment).

**Figure 4:**
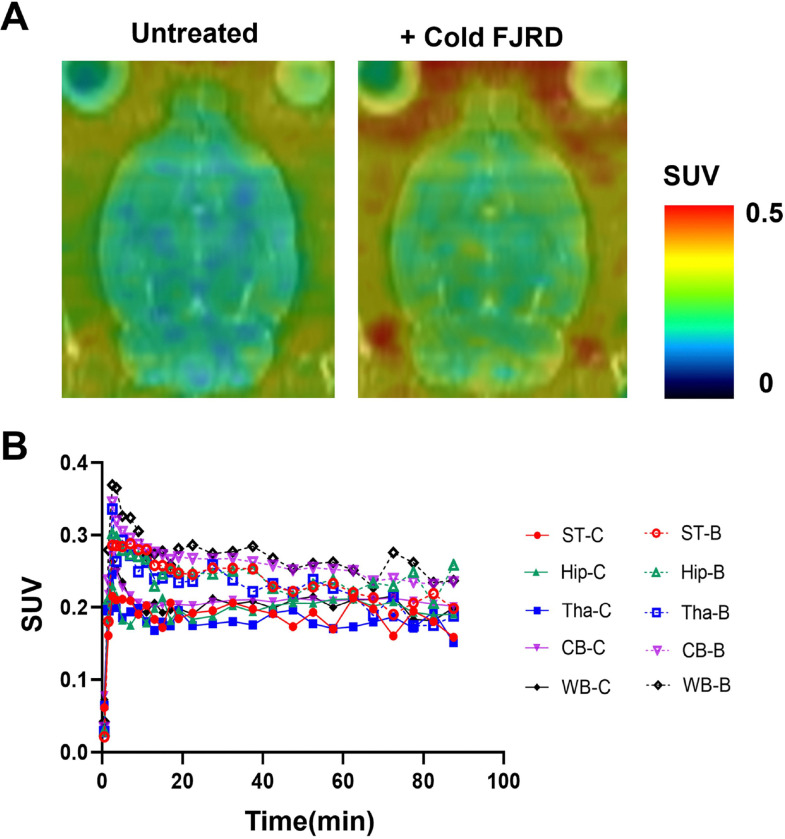
PET imaging of [^11^C]FJRD in rat brain. (A) Summation PET/MRI-fused images of [^11^C]FJRD (0–90 min) with or without pretreatment of cold FJRD (1 mg/kg). (B) Time-activity curves in the striatum (ST), hippocampus (Hip), thalamus (Tha), cerebellum (CB) and whole brain (WB). Data are expressed as SUV. -C: control; -B: block.

PET images and time-activity curves showed rapid biodistribution of [^11^C]FJRD in various organs of normal mouse with peak standardized uptake value (SUV) values of approximately 0.3–5 at the initial phase, followed by rapid washout except in the brain. Cold blocking with unlabeled FJRD did not show impact on its uptake in brain, heart, liver, lungs, while decreased kidney uptake by approximately 25% (Fig. 3). PET imaging was performed in the normal rat to investigate its uptake in brain subregions including the striatum, hippocampus, thalamus, and cerebellum. As results, no overt difference in brain permeability and washout was detected among brain subregions. Meanwhile, initial brain uptake with peak SUV value of approximately 0.4 and washout of [^11^C]FJRD in rat brain was similar with the observation in mouse. Pretreatment with cold FJRD increased brain uptake slightly, indicating no detectable specific binding (Fig. 4).

### *In-vitro* autoradiography

**Figure 5:**
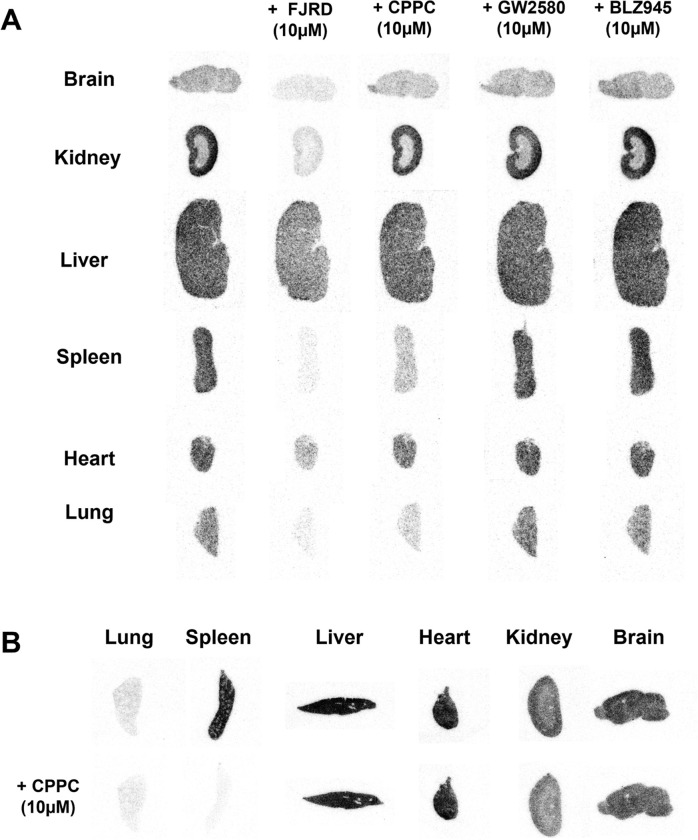
*In-vitro* autoradiography with [^11^C]FJRD and [^11^C]CPPC in the peripheral and central organs of healthy mouse. Representative *in-vitro* autoradiographs of [^11^C]FJRD (A) and [^11^C]CPPC (B) in the various organs from healthy mice in the absence or presence of non-radioactive compounds including FJRD, CPPC, GW2580 and BLZ945 at the concentration of 10 μM as indicated.

**Figure 6:**
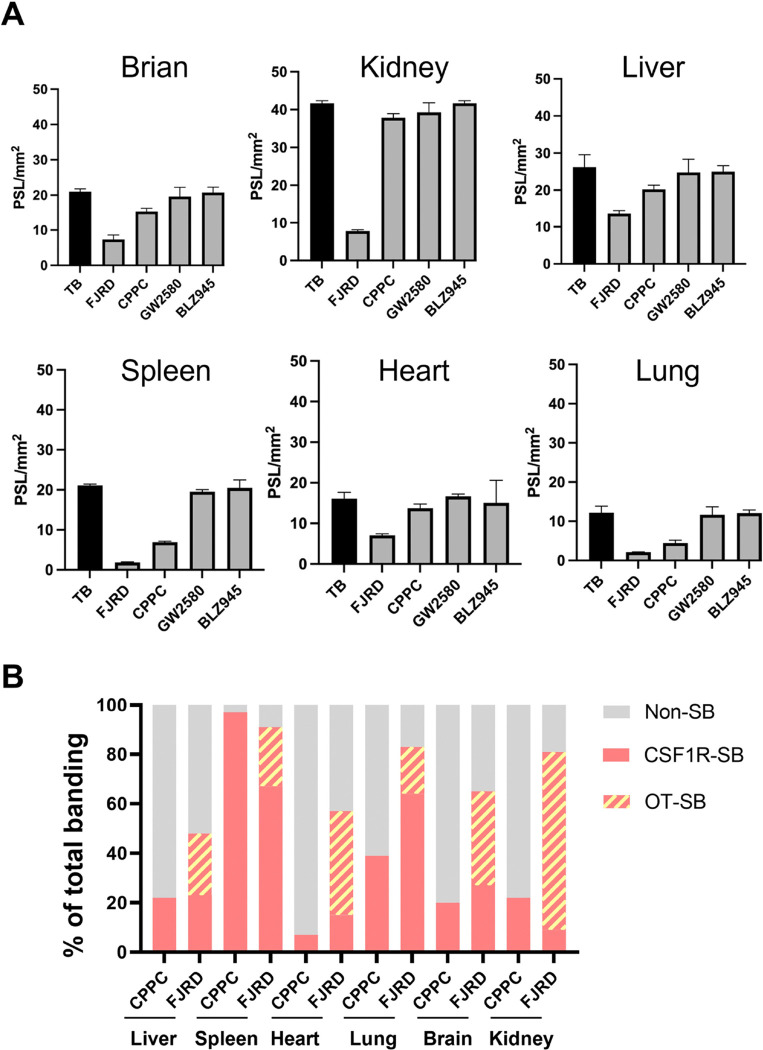
Quantitative analysis for *in-vitro* binding properties of [^11^C]FJRD and [^11^C]CPPC. (A) Inhibitory effects of various CSF1R inhibitors on [^11^C]FJRD *in-vitro* binding. Total binding (TB) is the binding of [^11^C]FJRD without the addition of any cold compound. N = 3 for each group. Data are expressed as mean ± SD. (B) The proportions of non-specific binding (non-SB), specific binding to CSF1R (CSF1R-SB) and specific binding to off-target (OT-SB) of [^11^C]FJRD (FJRD) and [^11^C]CPPC (CPPC) with respective total binding as 100%. Data from Figure 5.

*In-vitro* autoradiogram of [^11^C]FJRD showed that decreased radioactivities by the addition of cold (non-radioactive) FJRD and CPPC were 91% *vs* 67% in the spleen, 83% *vs* 64% in the lungs, 81% *vs* 9% in the kidneys, 65% *vs* 27% in the brain, 56% *vs* 15% in the heart and 48% *vs* 23% in the liver, respectively (Figs. 5 and 6A). Meanwhile, only faint decrease in radioactivity was detected in all tested organs in the presence of either GW2580 or BLZ945 (Figs. 5A and 6A).

**Figure 7:**
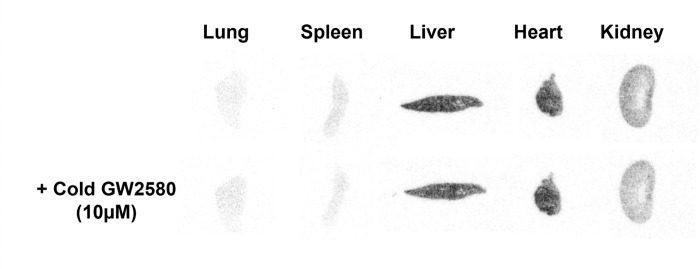
Representative *in-vitro* autoradiography with [^11^C]GW2580 in the peripheral and central organs of healthy mouse in the absence or presence of non-radioactive GW2580 (10 μM).

Given that CPPC is a well-known high-affinity compound for CSF1R, reduced radioactivity by the addition of cold FJRD and CPPC are considered whole specific binding and specific binding to CSF1R (CSF1R-SB), respectively. The gap of two binding is specific binding to off-target (OT-SB). The proportions of CSF1R-SB, OT-SB and non-specific binding (non-SB) for [^11^C]CPPC and [^11^C]FJRD with total binding as 100% were shown in Fig. 6B. CSF1R-SB of [^11^C]CPPC and [^11^C]FJRD were highest in the spleen with values of approximately 97% and 67%, respectively. The percentages of CSF1R-SB of [^11^C]FJRD are higher in the heart, lung and brain, compared to [^11^C]CPPC, suggesting its potential for more sensitive CSF1R-detectivity in these organs. However, there are also unignorable amount of OT-SB ranged from 19–72% in the tested organs (Fig. 6B). *In-vitro* autoradiography with [^11^C]GW2580 demonstrated that the addition of cold GW2580 did not decrease the binding of [^11^C]GW2580 in all organs above, suggesting no overt specific binding detectable in these organs under the normal condition (Fig. 7).

## Discussion

The present study has performed radiosynthesis of a novel *o*-aminopyridyl alkynyl derivative, [^11^C]FJRD, and evaluation of *in-vitro* and *in-vivo* binding properties in normal rodents. As results, there are great amount of specific binding clearly detected in the investigated peripheral and central organs. However, given that the addition of other three CSF1R inhibitors, CPPC, GW2580 and BLZ945 only showed partial or no blocking effects on [^11^C]FJRD binding, unignorable proportions are due to off-targeting bindings to unknown molecule(s) in these organs (Figs. 6 and 7). These findings provide a rationale to pay great attention to specificity for CSF1R as use radioactive *o*-aminopyridyl alkynyl derivative such as [^18^F]4 for CSF1R imaging^12^. There are several lines of CSF1R-PET tracer with different core chemical structures. Except for the compounds used in the present study, [^11^C]5 (3-((2-amino-5-(1-methyl-1*H*-pyrazol-4-yl)pyridin-3-yl)ethynyl)-*N*-(4-(2-fluoroethoxy)phenyl)-4-methylbenzamide) showed high-level specific binding in the normal rodent brain under *in-vitro* condition. However, low brain permeability greatly limits their application for neuroinflammation imaging in CNS disorders^13^. No available data on their specific binding to peripheral organs also make it unclear whether this ligand has potential as CSF1R imaging tracer for peripheral inflammation. The present study also confirmed overt specific binding of CPPC only detected in the spleen, similar with a previous publication ^10^. Partial blocking effect of CPPC on [^11^C]FJRD binding strongly indicates that these two compounds share a binding site on CSF1R molecule, which is different with that for GW2580 or BLZ945. Given that BLZ945 showed great blockage for [^11^C]5 under *in-vitro* condition, there are at least two binding sites on CSF1R protein for these CSF1R imaging tracers. Berend *et al* have reported a brain-permeable [^11^C]4 (3-((2-amino-5-(1-methyl-1*H*-pyrazol-4-yl)pyridin-3-yl)ethynyl)-*N*-(3-(2-fluoroethoxy)phenyl)-4-methylbenzamide) with a high affinity for CSF1R (IC_50_ = 12nM) and similar core structure of GW2580. Its brain uptake was significantly decreased by pretreatment with cold compound^14^. It is the only CSF1R PET tracer to date that demonstrates detectable specific binding in the living healthy mouse brain. However, the lack of the data on *in-vitro* binding of [^11^C]4 is disturbing, especially considering that [^11^C]GW2580 does not reveal its specific binding in all main organs under *in-vitro* autoradiographic condition. Further investigation is required for its binding property.

Some protein molecules such as VEGFR1, PDGFR-α, RET and c-Kit might provide off-target binding of [^11^C]FJRD, based on 3-((2-amino-5-(1-methyl-1*H*-pyrazol-4-yl)pyridin-3-yl)ethynyl)-*N*-(4-chlorophenyl)-4-methylbenzamide, an analogue of FJRD, inhibited their activities by 23.3–70.4% at 10nM concentration^14^. SYHA1813, a two-target inhibitor of VEGFR and CSF1R shares the core structure with FJRD and reveals very high affinity for VEGF receptors (VEGFRs) including VEGFR1, VEGFR2 and VEGFR3 with IC_50_ of 2.8, 0.3 and 4.3 nM, respectively^15^. These results imply VEGFRs as possible protein molecules providing the binding sites for [^11^C]FJRD. In the renal glomeruli, VEGFRs are highly expressed by both podocytes and glomerular endothelial cells^16^, in consistent with the present result of high off-target binding in kidneys (Fig. 6B).

Although off-target binding is a major concern, the present study provides several informative points for the development of promising CSF1R tracer in the future. (1) FJRD and CPPC share a binding site that is abundant in spleen, providing a positive-control material. (2) Higher *in-vitro* CSF1R-SB of [^11^C]FJRD is observed in heart, lung and brain compared to [^11^C]CPPC (Fig. 6B), and therefore indicate the potential for more sensitively detecting CSF1R in these organs.

## Conclusions

In the present study, we developed a novel ^11^C-labeled *o*-aminopyridyl alkynyl derivative, [^11^C]FJRD, as a potential CSF1R-PET tracer. Compared to currently available tracers, [^11^C]CPPC and [^11^C]GW2580, [^11^C]FJRD demonstrated enhanced sensitivity for detecting CSF1R in certain rodent organs. However, its modest brain-permeability and unignorable off-target binding highlight the need for further optimization to improve brain permeability and specificity for CSF1R.

## Methods

### General

All chemicals and organic solvents were purchased from Bidepharm and Energy Chemical, and used as supplied. ^1^H NMR spectroscopy and mass spectrometry (MS) were used to characterize isolated compounds. NMR spectra were recorded on either a Qone AS400 instrument 400 MHz or a Bruker Avance ☒, 400 MHz instrument. All ^1^H NMR experiments were reported in units, parts per million (ppm), and were measured relative to the signals for residual chloroform (7.28 ppm) or dimethyl sulfoxide (2.51 ppm) in the deuterated solvent, unless otherwise stated. The purities of the synthesized compounds were > 98%, as determined by analytical high-performance liquid chromatography (HPLC). Radio-HPLC was performed using a JASCO HPLC system (JASCO, Tokyo, Japan): effluent radioactivity was monitored using a NaI (Tl) scintillation detector system. Unless otherwise stated, radioactivity was measured using an IGC-3R Curiemeter (Hitachi Aloka Medical, Tokyo, Japan).

### The chemical synthesis of FJRD and its precursor for radiolabeling

The main steps of synthesis of compound FJRD are following previously reported routes ^11^ with slight modification. The chemical synthesis of intermediates as follow:

#### Methyl 3-((2-amino-5-bromopyridin-3-yl)ethynyl)-4-methylbenzoate (II)

Under an argon atmosphere, a mixture of methyl 3-iodo-4-methylbenzoate (276 mg, 1 mmol), 5-bromo-3-((trimethylsilyl)ethynyl)pyridin-2-amine (270 mg, 1 mmol), Pd(PPh_3_)_2_Cl_2_ (35 mg, 0.05 mmol ), CuI (19 mg,0.1 mmol), CsF (380 mg, 2.5 mmol), and Et_3_N (304 mg, 3 mmol) in MeCN (8.5 mL) was stirred at room temperature for 6 hours. After the reaction completed, the reaction mixture was concentrated *in vacuo*. The obtained residue was purified by column chromatography on silica gel (heptane/ethyl acetate = 8/1, v/v) to afford **II** as a yellow solid (303 mg, 88.2%). LC-MS, single peak, m/e, 346.2 (M + 1).

#### Methyl 3-((2-amino-5-(1-methyl-1*H*-pyrazol-4-yl)pyridin-3-yl)ethynyl)-4-methylbenzoate (III)

Under an argon atmosphere, a reaction vial was charged with **II** (270 mg, 0.8 mmol), methyl-4-(4,4,5,5-tetramethyl-1,3,2-dioxaborolan-2-yl)-1*H*-pyrazole (252 mg, 1.2 mmol), Pd(OAc)_2_ (18 mg, 0.08 mmol), X-phos (76 mg, 0.16 mmol), and K_2_CO₃ (276 mg, 2.0 mmol). A mixture of THF (5.5 mL) and H_2_O (1.4 mL) was then added to the vial. Then the mixture was stirred at 90°C for 6 hours. After the reaction completed, the reaction mixture was concentrated *in vacuo*. The reactant was purified by silica gel column chromatography (eluent: heptane/ethyl acetate = 2/1, v/v) to afford **III** as a yellow solid (221.5 mg, 80%). LC-MS, single peak, m/e, 347.1 (M + 1).

#### 3-((2-Amino-5-(1-methyl-1*H*-pyrazol-4-yl)pyridin-3-yl)ethynyl)-4-methylbenzoic acid (IV)

Compound **III** (200 mg, 0.58 mmol), and LiOH·H_2_O (61 mg, 1.44 mmol) were dissolved in a mixture of THF (5.5 mL), MeOH (0.5 mL), and H_2_O (1.4 mL). The reaction mixture was stirred at room temperature for 12 hours. Upon completion of the reaction, the mixture was cooled to 0°C, and the pH was adjusted to 4–5 by using 1 M HCl, leading to the formation of a crystalline solid. The mixture was stirred for an additional 30 minutes, and the precipitate was collected by filtration. The obtained solid was washed three times with water and dried to afford **IV** as a yellow solid (181 mg, 94% yield). LC-MS, single peak, m/e, 333.1 (M + 1).

#### 3-((2-Amino-5-(1-methyl-1*H*-pyrazol-4-yl)pyridin-3-yl)ethynyl)-*N*-(4-hydroxyphenyl)-4-Methylbenzamide (V-1, precursor for radiolabeling)

A mixture of compound **IV** (175 mg, 0.53 mmol), HATU (262 mg, 0.69 mmol), and DIPEA (206 mg, 1.59 mmol) in DMF (5 mL) was stirred at room temperature for 30 minutes, followed by the addition of *p*-toluidine (72 mg, 0.66 mmol). The reaction was allowed to proceed at room temperature for 4 hours. Upon completion, water was added to dilute the mixture, which was then extracted with ethyl acetate. The organic layer was collected, washed with a saturated saline solution, dried over Na_2_SO_4_, and concentrated *in vacuo*. The crude product was purified by silica gel chromatography (eluent: 100% ethyl acetate) to afford compound **V-1** as a yellow solid (184 mg, 79.6% yield). ^1^H NMR (400 MHz, DMSO*-d6*) δ 10.11 (s, 1H), 9.32 (s, 1H), 8.33 (d, *J* = 2.4 Hz, 1H), 8.28 (d, *J* = 1.8 Hz, 1H), 8.14 (s, 1H), 7.91 (td, *J* = 3.6, 1.9 Hz, 2H), 7.88 (d, *J* = 0.7 Hz, 1H), 7.63–7.57 (m, 2H), 7.52 (d, *J* = 8.1 Hz, 1H), 6.83–6.78 (m, 2H), 6.35 (s, 2H), 3.90 (s, 3H), 2.62 (s, 3H). LC-MS, single peak, m/e, 438.2 (M + 1).

#### 3-((2-Amino-5-(1-methyl-1*H*-pyrazol-4-yl)pyridin-3-yl)ethynyl)-*N*-(4-methoxyphenyl)-4-methylbenzamide (V-2, FJRD)

A mixture of compound **IV** (200 mg, 0.6 mmol), HATU (267 mg, 0.78 mmol) and DIPEA (233 mg, 1.8 mmol) in 5 mL DMF was stirred at room temperature for 30 minutes, followed by the addition of 4-aminophenol (71.4 mg, 0.58 mmol). The reaction was allowed to proceed at room temperature for 4 hours. Upon completion, water was added to dilute the mixture, which was then extracted with ethyl acetate. The organic layer was collected, washed with a saturated saline solution, dried over Na_2_SO_4_, and concentrated *in vacuo*. The crude product was purified by silica gel chromatography (eluent: 100% ethyl acetate) to afford **V-2** (FJRD) as a yellow solid (181 mg, 71.3%). ^1^H NMR (400 MHz, DMSO*-d6* ) δ 10.22 (s, 1H), 8.33 (d, *J* = 2.4 Hz, 1H), 8.30 (d, *J* = 1.8 Hz, 1H), 8.14 (s, 1H), 7.92 (dd, *J* = 9.6, 2.1 Hz, 2H), 7.88 (d, *J* = 0.7 Hz, 1H), 7.77–7.71 (m, 2H), 7.53 (d, *J* = 8.1 Hz, 1H), 7.03–6.96 (m, 2H), 6.35 (s, 2H), 3.90 (s, 3H), 3.81 (s, 3H), 2.62 (s, 3H). LC-MS, single peak, m/e, 424.1 (M + 1).

### Radiochemistry

Cyclotron-produced [^11^C]CO_2_ was introduced into 0.4 M LiAlH_4_ in anhydrous tetrahydrofuran (0.3 mL). After the tetrahydrofuran was evaporated, the remaining complex was reacted with 57% hydroiodic acid (0.3 mL) to yield [^11^C]CH_3_I. The [^11^C]CH_3_I was distilled with heating and transferred under a stream of N_2_ gas to a solution containing the precursor (1 mg) and NaOH (5 μL, 0.5 M) in *N,N*-dimethylformamide (DMF) (0.3 mL) at −15°C. After the trapping process was completed, the reaction mixture was heated to 80°C for 5 minutes. HPLC separation was conducted using a Capcell PAK UG80 C18 column (10 × 250 mm; Shiseido, Osaka, Japan) with a mobile phase of MeCN/H_2_O/Et_3_N (5/5/0.001, v/v/v) at a flow rate of 5.0 mL/min. The radioactive fraction corresponding to [^11^C]FJRD (tR = 9.2 min) was collected in a flask pre-loaded with Tween 80 (0.075 mL) and ethanol (0.3 mL). The collected fraction was then evaporated to dryness, redissolved in 3 mL of sterile normal saline containing 3.3% (v/v) Tween 80 and 0.8% (v/v) ascorbic acid, and filtered through a 0.22 μm Millipore filter (Billerica, MA, USA). The identity of [^11^C]FJRD was confirmed by co-injection with unlabeled FJRD on a reverse-phase analytical HPLC using a Capcell PAK UG80 C18 column (4.6 × 250 mm) with a mobile phase of MeCN/H_2_O/Et_3_N (55/45/0.001, v/v/v) at a flow rate of 1.0 mL/min (tR = 6.4 min). The molar activity was determined by comparing the measured radioactivity with the mass detected at UV (254 nm).

### Experimental animals

Three-month-old male C57Bl/6J mice and ten-week-old male Sprague-Dawley (SD) rats were procured from CLEA-Japan (Tokyo, Japan). Upon arrival, the animals were housed in the vivarium facilities at the National Institute of Radiological Sciences, where they were kept under standard laboratory conditions with controlled temperature and humidity. Food and water were provided ad libitum to ensure their well-being. The animals were acclimatized to the facility environment before the commencement of any experimental procedures, adhering to best practices in animal care and research protocols.

#### In - vitro autoradiographic analysis

*In-vitro* autoradiography was conducted following a previously published protocol with minor modifications^17^. Organ sections were first pre-incubated in PBS for 30 minutes to hydrated the tissue. Following this, the sections were incubated at room temperature for 30 minutes with incubation solution (3.8% BSA in PBS) containing radioactive tracer (5 nM [^11^C]FJRD or [^11^C]CPPC or [^11^C]GW2580) in the presence or absence of unlabeled compounds (10 μM) as indicated. After incubation, the sections were washed twice with PBS, each for 2 minutes, to remove unbound radioligands. The sections were then briefly immersed in distilled water for 10 seconds to remove any remaining salts. The tissues were subsequently air-dried under gentle warmth and then affixed to an imaging plate (BAS-MS2025; GE Healthcare). The exposure times on the imaging plate were optimized based on the specific requirements of each experiment. Radiolabeling was then detected by scanning the imaging plate using the BAS-5000 system (FUJIFILM, Tokyo, Japan), allowing for precise autoradiographic analysis of the radiotracer distribution within the sections. Regions of interest (ROIs) were carefully placed on whole organ. Total and non-specific binding were expressed by radioactivity in the absence and presence, of unlabeled compounds, respectively. Specific binding was calculated by total minus non-specific binding.

### Small-animal PET imaging

Mice or rats were anesthetized with 1.5–2.0% isoflurane and carefully positioned on the pre-heated scanner bed of small-animal PET scanners (Focus 220 for mouse and Inveon for rat; Siemens Medical Solutions Knoxville, TN, USA), as previously described ^17^. Following the positioning, mice or rats were intravenously injected with a solution of [^11^C]FJRD (37–55 MBq for mouse and 48–66 MBq for rat) immediately followed by a PET scan. Dynamic PET data were collected continuously over 60 minutes for mouse whole body and 90 minutes for rat brain scans. The energy window for detecting ^11^C emissions was set between 350–750 keV. Decay correction factors were applied, calculated from the start of the acquisition to a reference time point, which was defined as the initiation of the first acquisition in the first animal. Image reconstruction was performed using a maximum-a-posteriori algorithm to generate single-frame average images for qualitative analysis, and filtered backprojection with a 0.5-mm Hanning filter for generating dynamic images used in quantitative assessments. Volumes of interest (VOIs) were delineated on brain regions of rat and peripheral organs of mouse as indicated in the corresponding figures utilizing PMOD image analysis software (PMOD Technologies Ltd, Zurich, Switzerland).

## Data Availability

Data can be obtained upon request.

## Abbreviations

CSF1R: colony stimulating factor 1 receptor
PET: positron emission computed tomography
SUV: standardized uptake value
%ID/g: percentage of injection dose per gram tissue
X-phos: 2-Dicyclohexylphosphino-2’,4’,6’-triisopropylbiphenyl
THF: Tetrahydrofuran
DIPEA: *N,N*-Diisopropylethylamine
HATU: 2-(7-Azabenzotriazol-1-yl)-*N,N,N’,N*’-tetramethyluronium hexafluorophosphate
